# Effects of simulated acid rain on hydrochemical factors and microbial community structure in red soil aquifers†

**DOI:** 10.1039/d3ra08820k

**Published:** 2024-02-02

**Authors:** Yian Wang, Chao Long, Li Yin, Renlu Liu, Yonghui Liao, Genhe He, Zuwen Liu

**Affiliations:** a School of Life Science, Jinggangshan University Ji'an Jiangxi China yinli_voyage@126.com liurenlu89@163.com liaoyonghui1104@126.com liuzw@jxust.edu.cn nickowya@163.com hegenhe@jgsu.edu.cn; b School of Civil and Surveying & Mapping Engineering, Jiangxi University of Science and Technology Ganzhou Jiangxi China lc18832058300@163.com; c School of Hydraulic & Ecological Engineering, Nanchang Institute of Technology Nanchang China

## Abstract

Acid rain can lower the pH of groundwater and affect its hydrogeochemistry and microbial ecology. However, the effects of acid rain on the hydrogeochemistry and microbial ecology of red soil groundwater systems in southern China are poorly understood. Previous research had mainly investigated the sources and patterns of groundwater acidification, but not the microbial mechanisms that contribute to this process and their associations with hydrochemical factors. To address this knowledge gap, we conducted a soil column experiment to simulate the infiltration of acid rain through various filter materials (coarse, medium, and fine sand) and to examine the hydrochemical and microbial features of the infiltrate, which can reveal how simulated acid rain (pH 3.5–7.0) alters the hydrochemistry and microbial community composition in red soil aquifers. The results showed that the pH of the leachate decreased due to simulated acid rain, and that the leaching efficiency of nitrogen and metal ions was influenced by the particle size of the filter media. Illumina 16S rRNA gene sequencing revealed that the leachate was dominated by *Proteobacteria*, *Patescibacteria*, *Actinobacteria*, and *Acidobacteria*, with *Proteobacteria* accounting for 67.04–74.69% of the bacterial community and containing a high proportion of nitrifying and denitrifying bacteria. Additionally, several genera with heavy metal tolerance, such as *Burkholderia-Caballeronia-Paraburkholderia*, *Delftia*, *Methylversatilis*, *Aquicella*, and *Ralstonia*, were widely distributed in the leachate, indicating the strong adaptive capacity of the microbial population. A correlation analysis between the hydrochemical factors and the microbial community structure revealed that pH was the most influential factor, followed by NO_2_^−^-N, Fe, Al, Cu, Mn, and others. These results indicate that acidification modifies the hydrochemical conditions of the aquifer, creating an environment that is unfavorable for microbial growth and survival. However, some microorganisms may acquire resistance genes to cope with environmental changes.

## Abstract 2

Acid rain can lower the pH of groundwater and affect its hydrogeochemistry and microbial ecology.

## Graphical abstract

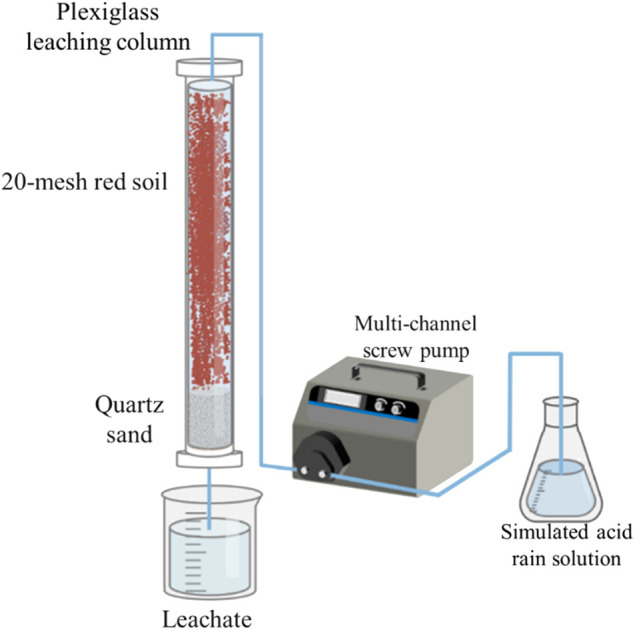

## Introduction

Red soil is a common type of acidic soil in the southern region of China. It has low base saturation and high leaching rate, which increase the H^+^ concentration in the soil and groundwater systems.^1,2^ Additionally, red soil areas often coincide with areas that receive high amounts of acid rain. The shallow groundwater in these areas is mainly recharged by atmospheric precipitation, which lowers the groundwater pH.^3,4^ Furthermore, red soil areas are major agricultural zones, where conventional farming practices apply large quantities of acid fertilizers and enhance the groundwater acidification.^5,6^ Groundwater acidification affects the aquifer pH, alters the hydrogeochemical cycle, and disrupts the hydrochemical equilibrium in the groundwater.^7,8^ It also increases the ion exchange capacity between water and rock, leading to higher levels of metal ions in the groundwater.^9,10^ Therefore, groundwater acidification not only modifies the hydrochemical characteristics of groundwater, but also elevates the bioavailability and toxicity of metal ions and impacts the groundwater ecosystem.

Microorganisms are the key components of the groundwater ecosystem, and their role in bioremediation of environmental pollutants and maintenance of ecosystem stability has been widely recognized.^11–13^ Previous studies have demonstrated that microorganisms influence the release and migration of arsenic, cadmium, copper, lead, aluminum and other metal ions in groundwater. Moreover, microorganisms can modulate the groundwater environment by altering the concentration of metal ions and other environmental factors.^14–17^ Therefore, the formation of acidified groundwater is likely to result from a series of complex interactions between microbiological and geochemical processes. However, most existing studies have focused on the distribution of acidic groundwater and its main causes,^18,19^ while the effects of acidified groundwater environmental factors on microbial communities have been largely overlooked. In addition, most of the current research on the relationship between microorganisms and acidification environment under acidic conditions is confined to the ocean or soil.^20–23^ Thus, the characteristics of microbial community structure in acidified groundwater and their responses to acidified environment remain unclear and warrant further investigation.

The pH of the environment is a critical factor influencing the sensitivity, activity, and composition of microbial communities.^24,25^ Molecular ecology methods can be used to analyze how microorganisms respond to acidification in groundwater.^26,27^ Li *et al.* simulated acid rain on forest soils and observed that acidification increased the fungal-to-bacterial ratio and reduced the soil respiration rate by 28.9% annually at pH 2.5, using high-throughput sequencing and phospholipid fatty acid analysis.^28^ Liu *et al.* reported similar results in a column experiment of simulated acid rain leaching of agricultural soil.^29^ However, some studies have suggested that acid rain may also stimulate microbial growth and metabolism by providing nitrogen and sulfur as metabolic substrates.^30,31^

This research aims to assess the impact of simulated acid rain (SAR) on the hydrogeochemistry and microbial diversity of red soil aquifers in China using soil column experiments. The specific objectives are to: (1) monitor the hydrogeochemical properties of red soil leachate, (2) identify the microbial community composition in the leachate, and (3) examine the correlation between the hydrogeochemistry and microbial diversity in the leachate under different acidification levels.

## Materials and methods

### Soil sampling

Soil samples were collected from a red earth region in Jiang'xi Province, eastern China (27°26′6′′ N, 115°33′48′′ E), which represents a typical acidic red soil. The surface soil of 0–80 cm depth was divided it into four layers: 0–20 cm, 20–40 cm, 40–60 cm, and 60–80 cm, and sampled each layer. The non-soil materials such as plant debris and stones from the pooled samples were removed. Some of the samples were stored in sterile self-sealing bags for moisture content determination, and air-dried, ground, sifted and stored the rest in a cool dry place for constructing an experimental model of layered soil column.

### Design and operation of the soil column simulation device

**Fig. 1:**
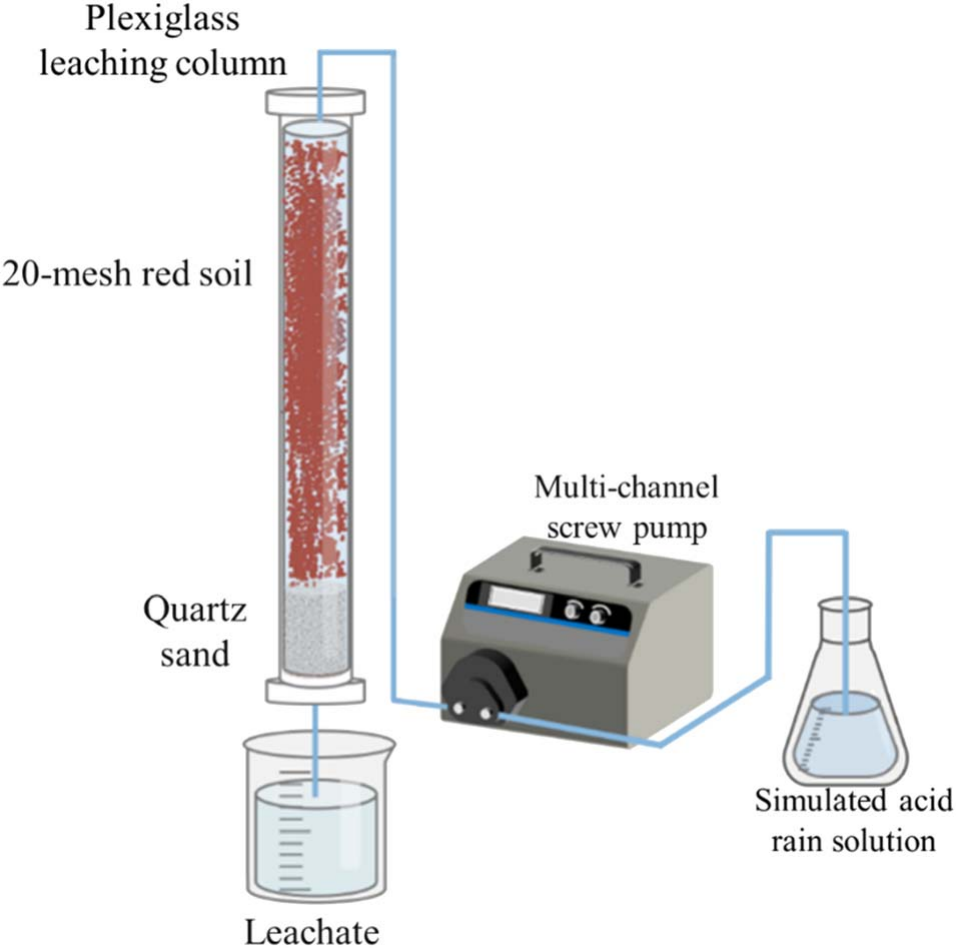
Schematic diagram of the soil column simulation device.

The experimental setup consisted of a plexiglass leaching column, as depicted in Fig. 1. The column had an inner diameter of 8 cm and a height of 100 cm. The bottom 20 cm of the column were filled with coarse sand (0.5–1 mm), medium sand (0.25–0.5 mm) and fine sand (0.1–0.25 mm) to simulate different aquifer media, respectively. The top 80 cm of the column were filled with air-dried red soils (four kinds of sub-layers based on their original depth at the sampling site: 0–20, 20–40, 40–60 and 60–80 cm) that were sieved through a 20 mesh screen. Each SAR treatment or the control with three experimental replicates. Thirty-six columns were set up and fixed on the stainless steel shelves. Glass fibers were placed on top of the surface soil in each column to prevent soil erosion and disturbance during the application of SARs.^32^ The columns were incubated at room temperature for 30 days before the experiment.

To prepare the SAR solutions, H_2_SO_4_ and HNO_3_ were mixed in a 5 : 1 ratio and then diluted with deionized water to achieve different pH levels. The SAR treatments had four pH levels of 7.0, 5.5, 4.5 and 3.5, corresponding to the current acidity and acidification trend of natural precipitations in the study area. The H_2_SO_4_ : HNO_3_ ratio in the SAR treatments was based on the SO_4_^2−^ : NO_3_^−^ ratio in natural precipitations in this region.^26,28^ Additionally, a control treatment used deionized water with a pH of 7.0.

The leaching amount of acid rain was estimated based on the annual rainfall in Jiang'xi Province, where the mean annual effective precipitation (after subtracting evaporation) is approximately 900 mm.^33,34^ Prior to the leaching experiment, high-purity water with a pH of 7.0 was used to flush from the bottom of the leaching column to expel the air and saturate the soil column. The acid rain leaching experiment commenced after 24 hours of equilibration. Each soil column was regulated by a multi-channel peristaltic pump (BT100M, Baoding Chuangrui pump Co., Ltd, Hebei, China) with a leaching rate of 6.3 mL h^−1^ for a total duration of 30 days. The leaching solutions were collected on the 30th day from the onset of the acid rain infiltration into the soil, and their hydrochemical properties, elemental composition and microbial community structure were analyzed.

### Leachate chemical analysis

The hydrochemical factors, such as nitrate nitrogen (NO_3_^−^-N), nitrite nitrogen (NO_2_^−^-N), ammonium nitrogen (NH_4_^+^-N), and pH were measured according to the standard methods described by APHA (1998). The levels of aluminum (Al), iron (Fe), manganese (Mn), copper (Cu) and cadmium (Cd) were analyzed by plasma optical emission spectrometer (ICP-OES, Optima 7000 DV, PerkinElmer, USA). The leaching rate was calculated by the following equation:
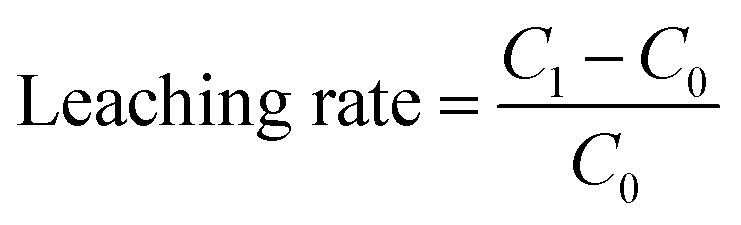
*C*_1_ was the substance concentration of after leaching; *C*_0_ was the substance concentration of before leaching.

### DNA extraction and high-throughput sequencing

The FastDNA Spin Kit for Soil (MP Biotechnology, Solon, OH, USA) was used to extract DNA from each sample following the manufacturer's protocol. The V3-V4 region of the 16S rRNA gene was amplified from the genomic DNA with 338F (ACTCCTACGGGAGGCAGCAG) and 806R (GGACTACHVGGGTWTCTAAT) primers.^35^ The 338F-806R specific primers with specific barcodes and high fidelity TrashStart FastPfu DNA polymerase (TransGen Biotech, China) were used for polymerase chain reaction (PCR) amplification. The PCR reaction mixture (20 μL) contained 4 μL of 5 × FastPfu Buffer, 2 μL of 2.5 mM dNTPs, 0.8 μL of 5 μM primers, 0.4 μL of FastPfu Polymerase and 10 ng of the extracted DNA template. The PCR thermal cycle profile consisted of: 2 min at 95 °C; 28 cycles of 30 s at 95 °C, 30 s at 61 °C, 45 s at 72 °C, and a final 10 min at 72 °C followed by cooling at 10 °C. An Illumina MiSeq platform was used for high-throughput sequencing (Majorbio Co. Ltd, Shanghai, China). Raw FASTQ files were demultiplexed and quality-filtered using QIIME based on the following criteria:^36^ (i) the reads were truncated at any site with an average quality score <20 over a 50 bp sliding window; (ii) sequences with overlaps longer than 10 bp were merged with mismatches ≤2 bp; (iii) the reads were demultiplexed according to barcodes (exact match) and primers (allowing 2 nucleotide mismatches), and reads containing ambiguous bases were removed. The UPARSE pipeline was used to cluster the remaining sequences into operational taxonomic units (OTUs) with 97% similarity.^37^

### Statistical analysis

The relative abundance of bacterial genera was used to classify them into two groups: major population (abundance > 1%) and rare population (abundance < 1%).^38,39^ A heatmap analysis was performed using *R* 4.2.0 to visualize the distribution of genera across the samples. The Spearman correlations between the hydrochemical parameters and the relative abundance of genera were calculated using SPSS 20.0 and considered significant at *p* < 0.05.

## Results and discussion

### Leachate hydrochemical characteristics

**Fig. 2:**
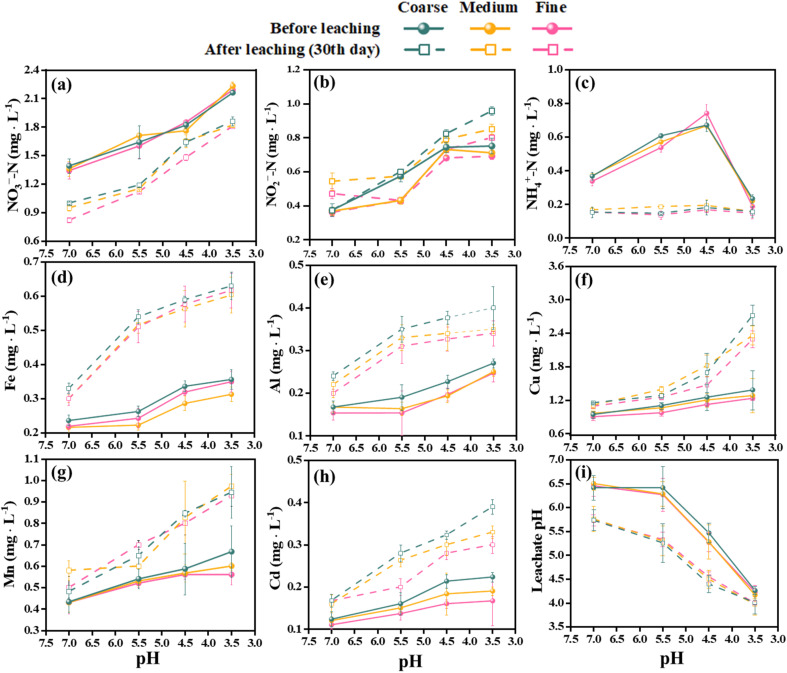
The pH of the leachate changed the hydrochemical characteristics after 30 days of SAR leaching. The initial values are shown by circles, while the final values are shown by rectangles.

The effects of SAR on the chemical parameters and heavy metal leaching of different aquifer media were investigated using leaching columns. Fig. 2 shows the variations of pH, nitrogen species and metal ions in the leachate of coarse, medium, and fine quartz sand. The initial (before leaching) pH of the leachate was higher than that of the after leaching for all media. However, after 30 days of leaching, the pH of the leachate decreased in all cases. The pH values were positively related to the particle size of the media, with the larger particles having higher pH values of leachate. Previous studies have generally agreed that acid rain can deplete soil sources of base cations and lower the pH of the soil vadose zone and contributes to the acidification of groundwater sources.^40,41^ This is especially true in the red soil regions of southern China, which have distinctive properties such as acidity, viscosity, and low permeability. The low-level acid rain had a negligible impact on aquifer acidification, while high-level acid rain significantly increased the risk of groundwater contamination, which may be due to long-term acid rain promoting the leakage of heavy metals.^42,43^ The bigger particle sizes lead to wider pores, allowing higher flow rates; the smaller the particle size of the aquifer medium, the higher the buffering capacity of the soil, which may be related to the difference in porosity.^44^

Moreover, the soil vadose zone had a strong buffering effect on acid rain, but there were large variations in the pH and its change trend among different aquifers under different pH treatments of SAR (Fig. 2i). The NO_2_^−^-N concentrations in the leachate increased significantly after 30 days, ranging from 5.97% to 52.82%. On the other hand, the NH_4_^+^-N and NO_3_^−^-N concentrations decreased by 20.86% to 77.30% and 6.25% to 54.84%, respectively. The NH_4_^+^-N concentrations of the after leaching solution were not affected by the pH values of the SAR. The acidification of the soil enhanced the leaching of nitrogen to the groundwater, causing groundwater nitrogen contamination, as documented by previous studies.^45^ The present study also revealed negative correlations between pH and three nitrogen concentrations. Furthermore, the filter media influenced the nitrogen leaching, with the larger particle sizes having higher leaching efficiency.

**Fig. 3:**
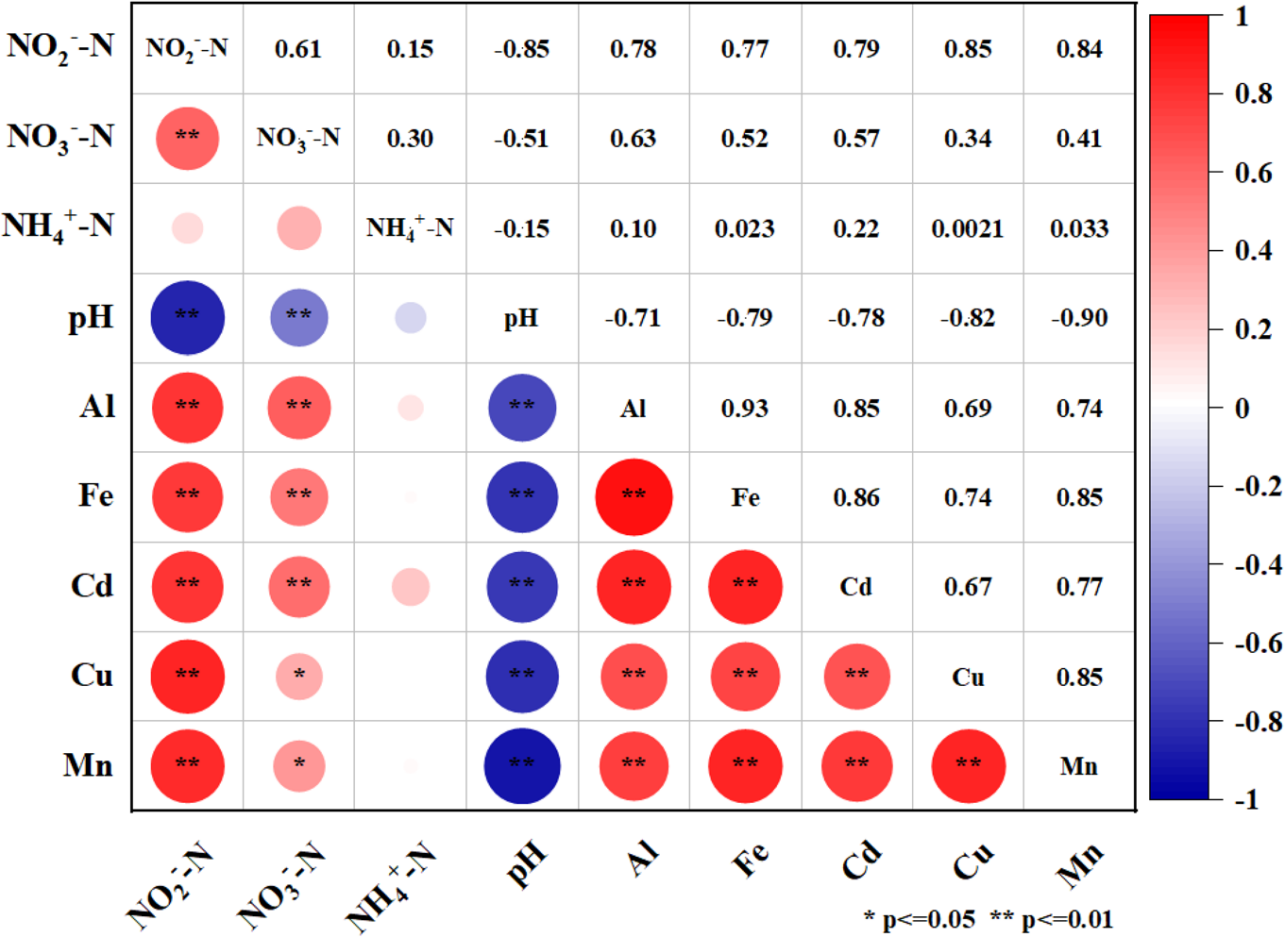
Spearman correlation matrix of hydrochemical characteristics.

The leachate from the waste showed increasing concentrations of heavy metals over time. Fe, Al, and Cd had the highest leaching rates among the metals, with mean values of 79.82%, 60.35%, and 59.50%, respectively, after 30 days in all media. Mn had the lowest mean leaching rate of 36.13%. The leaching rates of metals also varied significantly under different acidification conditions in 30 days. The maximum leaching rates for Fe and Al were observed at pH 5.5 and fine sand, with values of 131.84% and 102.61%, respectively. The maximum leaching rate for Cd was 81.97% at pH 4.5 and medium sand, which was 48.64% higher than at pH 7.0 and 8.29% higher than at pH 3.5. The maximum leaching rates for Cu and Mn were 97.10% (coarse sand) and 66.07% (fine sand) at pH 3.5, respectively, which were 74.76% and 49.79% higher than at pH 7.0 (Fig. 2). Moreover, the Spearman rank correlation analysis (Fig. 3) revealed significant negative correlations between pH and Fe (*r* = −0.79, *P* < 0.01), Al (*r* = −0.71, *P* < 0.01), Mn (*r* = −0.90, *P* < 0.01), Cu (*r* = −0.82, *P* < 0.01) and Cd (*r* = −0.78, *P* < 0.01). These metals also correlated positively with the leaching time and the particle size of the aquifer media, indicating that continuous acidification could enhance the leaching rate of heavy metal ions and pose a potential risk to soil and groundwater quality. The pH strongly influenced the leaching rates of these heavy metal ions, and continuous acidification could increase their leaching potential and pose a risk to soil and groundwater quality.^43^ In addition, Fe, Al, Mn, Cd and Cu had positive correlations with each other (*r* values from 0.67 to 0.93, *P* < 0.01), suggesting that they might have similar sources or associations.^7,19^

### Microbial diversity and community structure

**Fig. 4:**
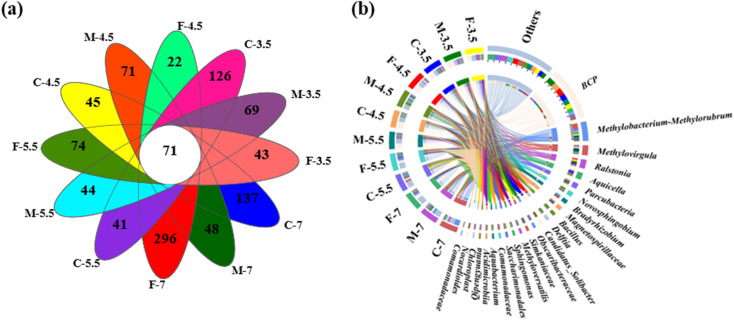
(a) Venn diagram showing the OTUs of the microbial community at different sites; (b) Circos diagram illustrating the relationship between groundwater samples and microbial taxa at the genus level (top 24 genera). The letters C, M, F indicate coarse, medium, and fine sand, respectively; the number 7, 5.5, 4.5, and 3.5 denote pH of leachate, which were consistent below.

The microbial names of the samples from coarse, medium, and fine sand at different pH levels are provided in the ID of Table S1.† The number of sequences per sample ranged from 52,234 to 73,054 (Table S1†). A Venn diagram showed that all samples shared 71 OTUs, while some OTUs were specific to certain samples (*e.g.*, 296 in sample F7 and 22 in sample F4.5) (Fig. 4a). Rarefaction curves and coverage estimates suggested that the sequencing depth adequately captured the bacterial diversity (>99%). The Shannon and Simpson indices, which measured the alpha diversity of the microbial community, showed that the microbial diversity decreased and the microbial uniformity increased as the pH decreased, respectively (Table S1†).

The microbial community composition differed significantly among the twelve soil columns, as Fig. S2† shows. At the phylum level, *Proteobacteria*, *Patescibacteria*, *Actinobacteria*, *Firmicutes* and *Acidobacteria* were the most abundant classes (>1%) in all leachate samples. *Proteobacteria* was the dominant class, comprising 67.07%–74.69% of the total sequences. *Patescibacteria*, *Actinobacteria*, *Firmicutes* and *Acidobacteria* had relative abundances of 4.83–6.45%, 3.58–8.43%, 2.72–17.48%, and 2.56–3.87%, respectively. *Firmicutes* was the second most abundant class in M4.5, with a relative abundance of 17.48%. These phyla have also been reported as dominant in acidified soil, sediment and water.^46,47^ Moreover, continuous H^+^ input did not cause significant changes in microbial community structure at the phylum level.

**Fig. 5:**
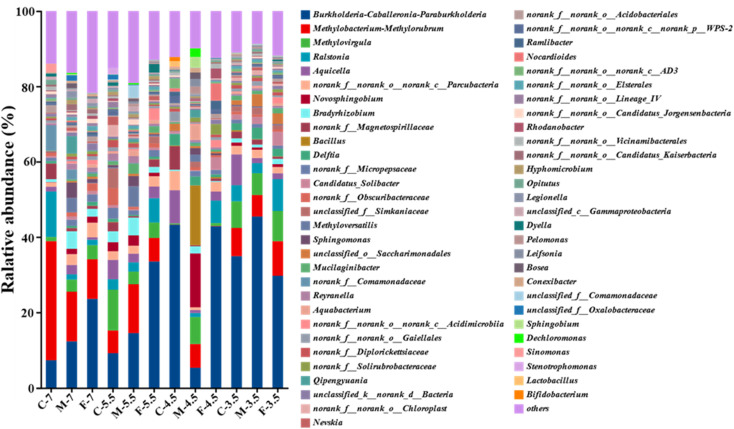
Composition of microbial in leachate at the genera level.

A Circos plot showed the major genera detected (>3%) (Fig. 4b); the top ten genera were *Burkholderia-Caballeronia-Paraburkholderia*, *Methylobacterium-Methylorubrum*, *Methylovirgula*, *Ralstonia*, *Aquicella*, *Parcubacteria*, *Novosphingobium*, *Bradyrhizobium*, *Magneto-spirillaceae* and *Bacillus*. *Burkholderia-Caballeronia-Paraburkholderia* (Burkholderiaceae family) had the highest relative abundance in F7 (23.6%), M5.5 (14.5%), F5.5 (33.5%), C4.5 (43.3%), F4.5 (42.9%), C3.5 (34.9%), M3.5 (45.43%) and F3.5 (29.7%), while *Methylobacterium-Methylorubrum*, *Methylovirgula* (Beijerinckiaceae family), and *Bacillus* (Bacillaceae family) prevailed in C7 (31.6%), and M4.5 (15.9%), respectively (Fig. 5). Heavy metal ions released by SAR increased the selection pressure on microbial communities, leading to the emergence of heavy metal tolerant bacteria in contaminated water or soil.^48^*Burkholderia-Caballeronia-Paraburkholderia* became the dominant population with increasing acidification. Some studies have shown nitrogen fixation and nitrification functions of *Burkholderia* sp. in the plant rhizosphere.^49^ In addition, other heavy metal tolerant bacterial groups, such as *Aquicella*, *Delftia*, *Bacillus* and *Saccharimonadales*, which have been reported in heavy metal polluted soils, sediments and aquifers, were widely distributed in this study and exhibited strong acid resistance.^50,51^ The adaptation mechanisms of these microorganisms to heavy metal pollution involved biosorption, biological precipitation, extracellular precipitation and chelation.^52^

In addition, metal ions may act as electron donors when organic matter is absent, as in the Feammox process. This anaerobic process couples ammonium oxidation and iron(iii) reduction, yielding nitrogen, nitrate and nitrite.^53^ This process could also take place in our simulated reactor, which could account for the decrease of NH_4_^+^-N and the increase of NO_2_^−^-N in the leachate. The correlation between NO_2_^−^-N, NO_3_^−^-N and Fe was much stronger than that between NH_4_^+^-N and Fe (Fig. 3), which also supported this hypothesis. However, there was no correlation between nitrate nitrogen and Fe, Al, Mn, Cu or Cd, which may suggest that metal ions inhibited the activity of nitrate bacteria.^54^ This also implies that the nitrification process in our system was severely impaired by acidification, which is in agreement with some studies on the effect of acidification on sludge nitrification characteristics.^55,56^

The genus-level microbial communities (top 20 in abundance) in different SAR leachates were compared by a multi-group difference test based on Kruskal Wallis h test (Fig. S3†). The test revealed that the abundance of *Burkholderia-Caballeronia-Paraburkholderia* and four other bacterial groups (*Aquicella*, *Delftia*, *Candidatus-Solibacter*, *Saccharimonadales*) increased as the SAR pH decreased. Conversely, the abundance of *Methylobacterium-Methylorubrum* (18.52–2.24%), which ranked second, and eight other genera (*Ralstonia*, *Parcubacteria*, *Bradyrhizobium*, *Magnetospirillaceae*, *Micropepsaceae*, *Obscuribacteraceae*, *Methyloversatilis*, *Sphingomonas*) decreased with the SAR pH. A survey of aquifer microbes in the Golmud area showed that the abundance of Alpha- and Beta-*proteobacteria* decreased with the aquifer grain size, while Gamma-*proteobacteria* increased. The main factor contributing to this result was pH change, which could alter the valence and bioavailability of elements by affecting their speciation.^57,58^ However, Liao *et al.* reported that the relative abundance of Alpha-*proteobacteria*, Gamma-*proteobacteria*, *Deltaproteobacteria* and *Bacteroidetes* increased with the soil aggregate size.^59^ The aquifer porosity determines the surface area-to-volume ratio of the aquifer material. A higher surface area facilitates chemical adsorption and microbial attachment, while a lower pore size restricts water flow, which limits the exchange of materials and energy between microorganisms and aqueous solutions, thus affecting the microbial community structure.^60^ The relative abundance of *Burkholderia-Caballeronia-Paraburkholderia*, a group of Gamma-*proteobacteria*, was negatively correlated with grain size in aquifer material at pH 7 and 5.5 (Fig. 5). This may reflect the higher porosity and specific surface area of the finer particles, or the pH variation induced by the grain size. Therefore, the dominant microbial community could be influenced by either factor.

### Correlations between microbial community and leachate hydrochemical characteristics

**Fig. 6:**
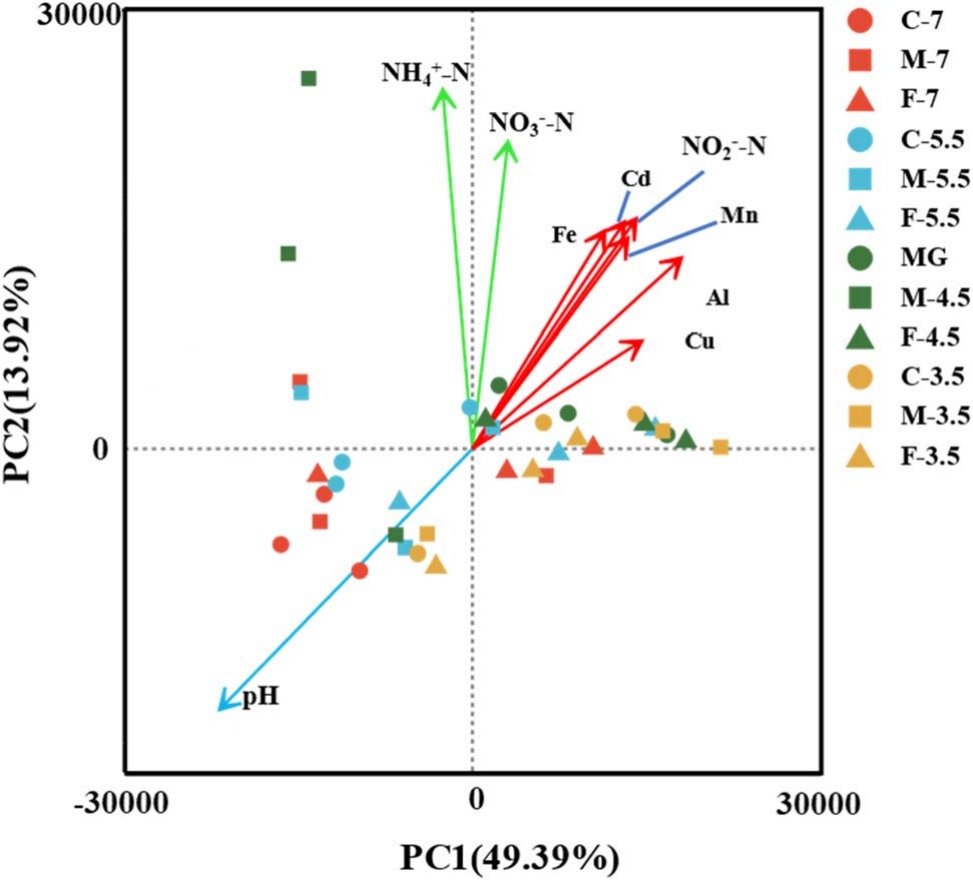
PCA analysis of the hydrochemical characteristics and the microbial community of the samples at the OTU level. The hydrochemical groups I, II, and III are indicated by green, red, and blue arrows, respectively.

To investigate how the key hydrochemical parameters affect the microbial community composition in the leachate samples with different pH and particle size, we performed Principal Component Analysis (PCA) and plotted the scores of pH, NH_4_^+^-N, NO_2_^−^-N, NO_3_^−^-N, Fe, Al, Cd, Cu, Mn (Fig. 6). The PCA results showed that the microbial community composition was influenced by three groups of hydrochemical parameters: (i) pH (group I), (ii) NH_4_^+^-N and NO_3_^−^-N (group II), and (iii) Mn, Fe, Al, Cd, Cu and NO_2_^−^-N (group III).

**Fig. 7:**
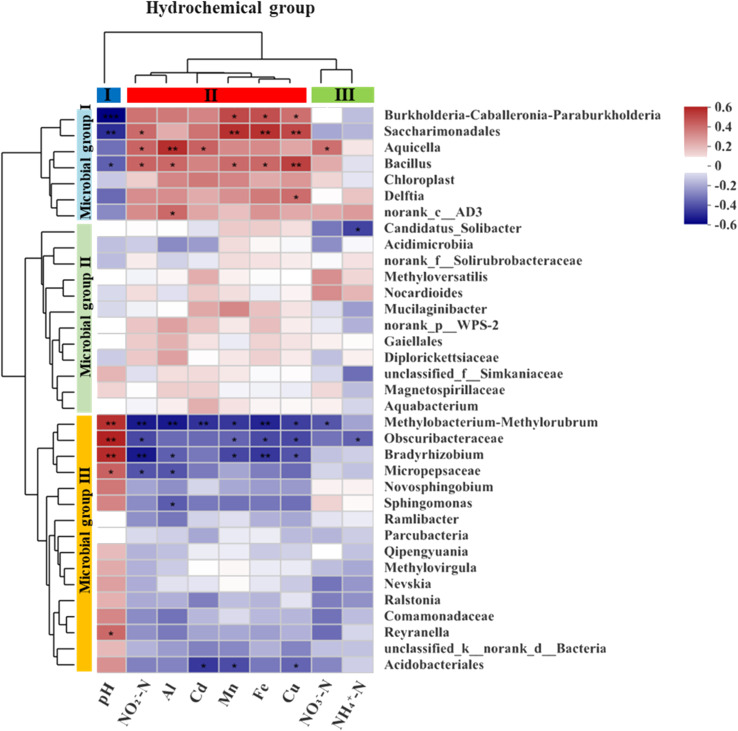
The heatmap of the Spearman correlation between the hydrochemical factors and the relative abundances of the dominant genera (top 35) in the samples.

A heatmap based on Spearman correlation coefficients was used to visualize the association between the most abundant microbial genera (top 35) and the three groups of hydrochemical factors (Fig. 7). The microbial genera were phylogenetically clustered into three groups. Microbial group I had significant positive correlations with pH (*p* < 0.05) for *Burkholderia-Caballeronia-Paraburkholderia*, *Saccharimondales* and *Bacillus*, and with NO_3_^−^-N (*p* < 0.05) for *Aquicella*. Microbial group II showed negative correlation (*p* < 0.01) with NH_4_^+^-N for *Candidatus_Solibacter*. Microbial group III correlated with various geochemical parameters involved in N cycling (4 genera), Al cycling (4 genera), Mn cycling (4 genera), Cu cycling (4 genera), Fe cycling (3 genera) and Cd cycling (2 genera).

The availability of nutrients and metabolic pathways, as well as the environmental conditions, affect the composition and dominance of microbial communities. Hydrochemistry (*e.g.*, pH, metal ions, redox state) is one of the environmental factors that has a significant influence on the distribution and diversity of microorganisms.^61,62^*Burkholderia-Caballeronia-Paraburkholderia*, which is a diazotrophic bacterium with heavy metal tolerance and nitrate reduction ability,^63–65^ had a positive correlation with metal ion concentration (*p* < 0.05) and a negative correlation with nitrate. *Aquicella* also showed a positive correlation with metal ion concentrations, especially with Al (*r* = 0.49, *p* < 0.01) and Cd (*r* = 0.36, *p* < 0.05). *Bradyrhizobium*, a rhizobial symbiont of legumes with nitrogen fixation activity,^66,67^ was correlated with nitrogen elements (*p* < 0.01). *Bacillus*, a common soil inhabitant belonging to the phylum *Firmicutes*, is a nitrifier that can oxidize ammonia and nitrite. A *Bacillus* strain isolated from electroplating wastewater sludge had tolerance to multiple heavy metals, such as Mn, Ni, Cd, Co, and Cr.^68^*Bacillus* had a significant positive correlation with Al, Cd, Mn, Fe, and Cu (Fig. 7). *Delftia* can tolerate Cd and reduce its accumulation in plants when inoculated in soil.^69^*Ralstonia* also exhibits Cd tolerance characteristics.^70^*Novosphingobium*, which is frequently found in soils and sediments, can resist Mn, Pb, Cr, Cd, Cu and degrade hydrocarbons and remediate petroleum-contaminated soils.^71^*Methyloversatilis* are mesophilic and facultative anaerobes that use nitrate as an electron acceptor under anoxic conditions. They can utilize iron or hydrogen as electron donors,^72^ and in this study they were negatively correlated with iron content and pH. Based on the above description, the dominant genera in the microbial communities could be classified into three groups: (i) acidophilic and heavy metal-tolerant bacteria, (ii) bacteria that can use metal elements such as iron and manganese as electron donors for nitrification and denitrification processes, and (iii) root-associated nitrogen-fixing bacteria.

## Conclusion

In this study, we investigated how SAR with varying pH affected the hydrochemical factors and microbial community structure of a red soil aquifer with different filter media (coarse, medium and quartz fine). SAR lowered the pH of the leachate and increased its acidity over time. The filter media influenced the leaching of NO_2_^−^-N, with larger particles resulting in higher leaching efficiency. Continuous acidification could alter the soil nitrogen cycle, release heavy metals, and worsen the soil and groundwater pollution. The pH had a negative correlation with the concentrations of three nitrogen forms and heavy metal ions, while NH_4_^+^-N and NO_2_^−^-N had a positive correlation with Fe, Al, Mn, Cu and Cd. The microbial community structure adapted to the acidified conditions by changing its composition to include heavy metal resistant bacteria, nitrifying-denitrifying bacteria, and root azotobacter. pH was the most influential hydrochemical factor on the microbial community composition and diversity, followed by NH_4_^+^-N, NO_3_^−^-N, and others. The acidification could change the hydrochemical conditions of the aquifer, create environmental stress for the microbial community, and also promote the emergence of more resistant microorganisms through gene transfer mechanisms to cope with environmental changes.

## Data availability

The authors confirm that the data supporting the findings of this study are available within the article and/or its ESI.†

## Author contributions

Yi'an Wang: Conceptualization, methodology, data curation, funding acquisition, writing – original draft, review & editing. Chao Long: investigation, data curation, methodology, writing – original draft. Li Yin: methodology, investigation. Renlu Liu: supervision, investigation. Yonghui Liao: supervision, investigation. Genhe He: conceptualization, funding acquisition, supervision, writing –original draft & review. Zuwen Liu: supervision, conceptualization, investigation, funding acquisition.

## Conflicts of interest

The authors declare that they have no known competing financial interests or personal relationships that could have appeared to influence the work reported in this paper.

## Supplementary Material

RA-014-D3RA08820K-s001
