# Supplementary material for: Effects of simulated acid rain on hydrochemical factors and microbial community structure in red soil aquifers
Source: RSC Adv. 2024 Feb 2;14(7):4482–91. doi: 10.1039/d3ra08820k (PMC10835706; doi:10.1039/d3ra08820k)
Supplement: RA-014-D3RA08820K-s001 [file RA-014-D3RA08820K-s001.pdf]

Electronic Supplementary Material (ESI) for RSC Advances.
This journal is © The Royal Society of Chemistry 2024

Supplementary Material

Effects of simulated acid rain on hydrochemical factors and microbial community structure in red soil aquifers

Yian Wang[1†], Chao Long[2†], Li Yin[1], Renlu Liu[1], Yonghui Liao[1], Genhe He[1*], Zuwen Liu[1,2,3]

[1]School of Life Science, Jinggangshan University, Ji'an, Jiangxi, China

[2]School of Civil and Surveying & Mapping Engineering, Jiangxi University of Science and Technology, Ganzhou, Jiangxi, China

[3]School of Hydraulic & Ecological Engineering, Nanchang Institute of Technology, Nanchang, China

†These authors (Yian Wang, nickowya@163.com; Chao Long, lc18832058300@163.com) contributed equally to this work and share first authorship.

* **Correspondence:** Genhe He (hegenhe@jgsu.edu.cn)

Tables

Table S1. The alpha diversity of microbial community in 12 soil columns simulated acid rain leaching.

pH	Diameter grade	ID	Reads	OUT	Sobs	Shannon	Simpson	Ace	Chao	Coverage
**7.0**	**Fine**	**F7**	54111	707	699.	3.307	0.147	880.59	879.98	0.995
	**Medium**	**M7**	68337	730	654	3.829	0.069	869.12	885.41	0.995
	**Coarse**	**C7**	73054	878	786	4.137	0.090	1040.4	1047.35	0.995
**5.5**	**Fine**	**F5.5**	67213	778	707	3.897	0.049	1079.09	1011.45	0.994
	**Medium**	**M5.5**	66332	741	679.5	3.606	0.087	1053.28	975.117	0.995
	**Coarse**	**C5.5**	59293	676	626.5	2.867	0.212	1103.93	947.59	0.994
**4.5**	**Fine**	**F4.5**	52234	549	535	2.755	0.224	711.66	714.31	0.996
	**Medium**	**M4.5**	72347	523	469	2.766	0.202	598.12	590.09	0.997
	**Coarse**	**C4.5**	64418	830	536	2.060	0.212	1059.94	714.64	0.991
**3.5**	**Fine**	**F3.5**	60469	870	875	2.969	0.222	1064.88	1041.81	0.995
	**Medium**	**M3.5**	62070	740	684	3.018	0.219	854.26	838.72	0.996
	**Coarse**	**C3.5**	61538	708	669	3.422	0.116	778.40	764.85	0.996

Figures

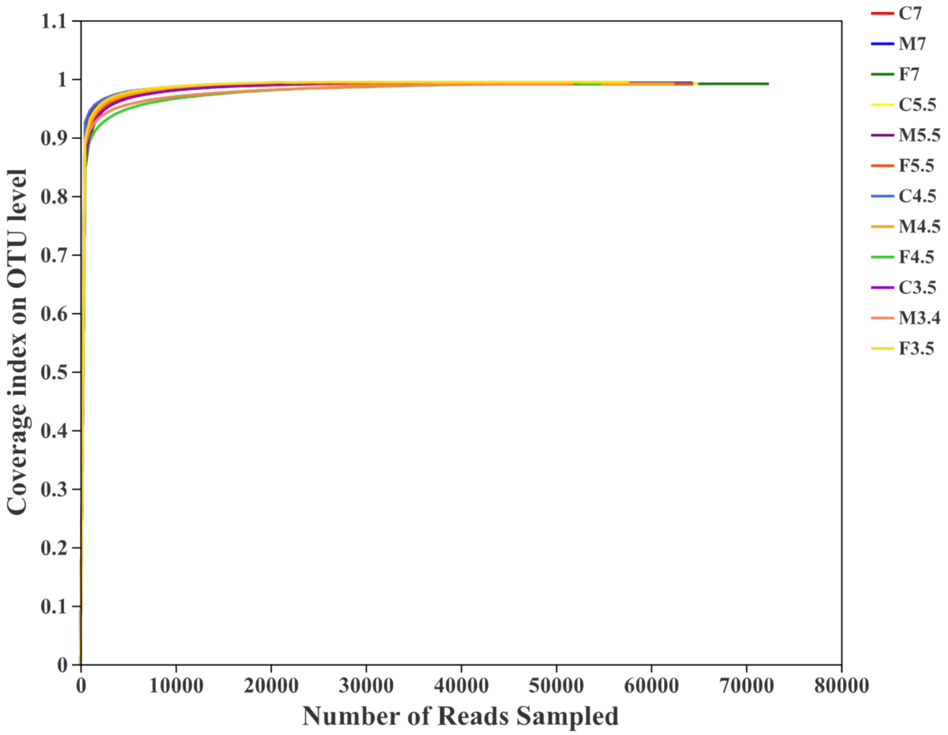

Fig. S1 Coverage rarefaction curves plot.

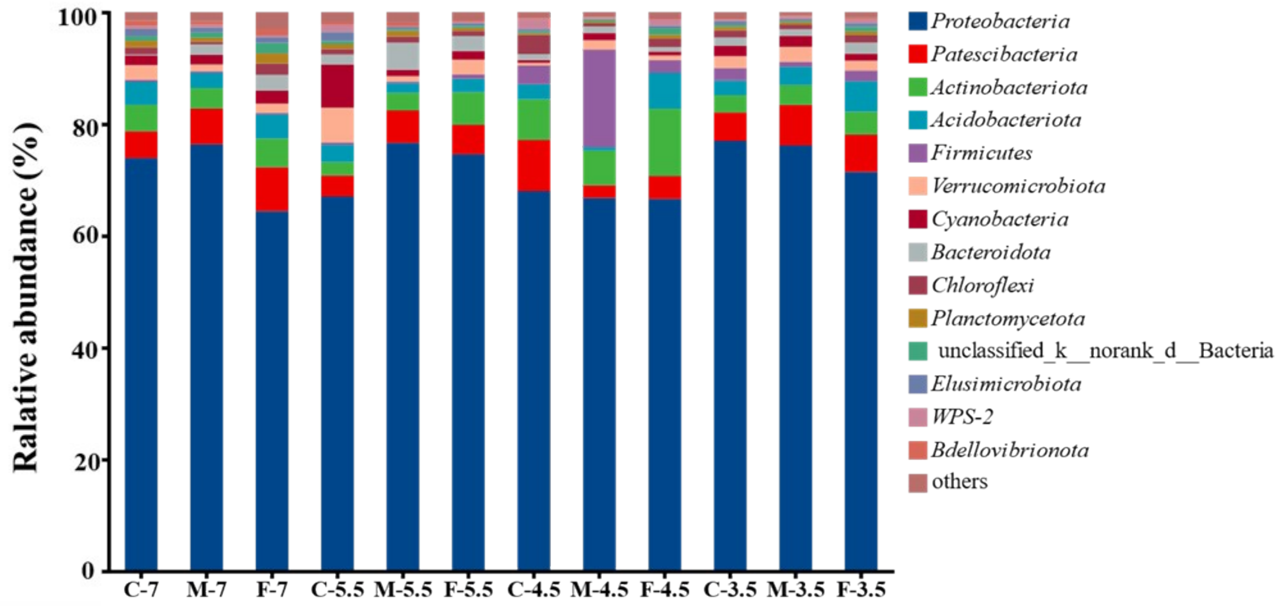

Fig. S2 Relative abundance of the microbial in leachate at the phylum level.

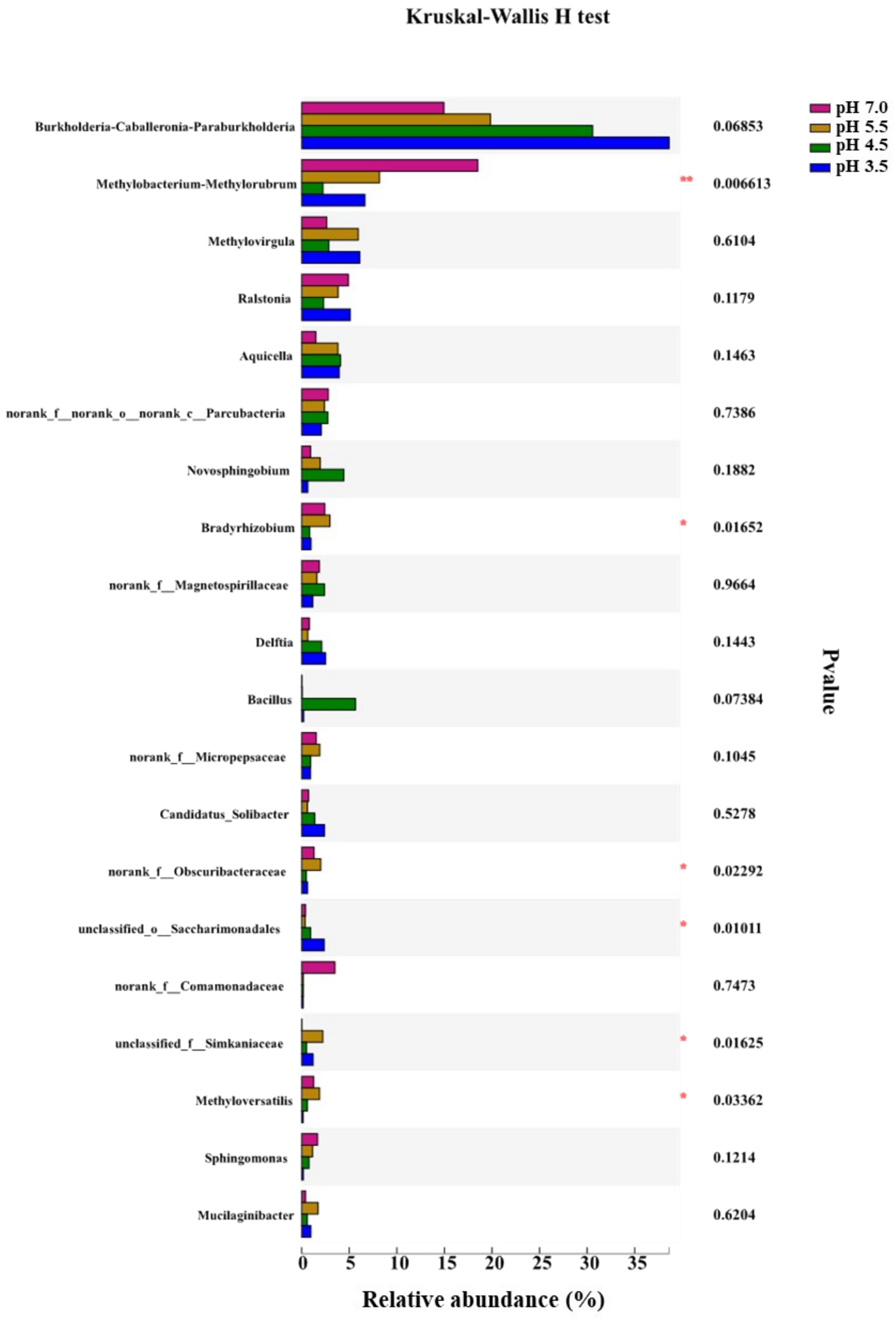

Fig. S3 A Kruskal-Wallis H test of the differences in microbial community composition at the genus level among different pH treatments. (Top 20; * p<=0.05; ** p<=0.01).
